# Breaking In: Human Metapneumovirus Fusion and Entry

**DOI:** 10.3390/v5010192

**Published:** 2013-01-16

**Authors:** Reagan G. Cox, John V. Williams

**Affiliations:** 1 Vanderbilt University School of Medicine, Department of Pathology, Microbiology and Immunology, 1161 21st Ave. S., Nashville, TN 37232, USA; E-Mail: reagan.j.cox@vanderbilt.edu; 2 Vanderbilt University School of Medicine, Departments of Pediatrics and Pathology, Microbiology, and Immunology, 1161 21st Ave. S., Nashville, TN 37232, USA

**Keywords:** metapneumovirus, fusion protein, paramyxovirus, integrin

## Abstract

Human metapneumovirus (HMPV) is a leading cause of respiratory infection that causes upper airway and severe lower respiratory tract infections. HMPV infection is initiated by viral surface glycoproteins that attach to cellular receptors and mediate virus membrane fusion with cellular membranes. Most paramyxoviruses use two viral glycoproteins to facilitate virus entry—an attachment protein and a fusion (F) protein. However, membrane fusion for the human paramyxoviruses in the *Pneumovirus* subfamily, HMPV and respiratory syncytial virus (hRSV), is unique in that the F protein drives fusion in the absence of a separate viral attachment protein. Thus, pneumovirus F proteins can perform the necessary functions for virus entry, *i.e.*, attachment and fusion. In this review, we discuss recent advances in the understanding of how HMPV F mediates both attachment and fusion. We review the requirements for HMPV viral surface glycoproteins during entry and infection, and review the identification of cellular receptors for HMPV F. We also review our current understanding of how HMPV F mediates fusion, concentrating on structural regions of the protein that appear to be critical for membrane fusion activity. Finally, we illuminate key unanswered questions and suggest how further studies can elucidate how this clinically important paramyxovirus fusion protein may have evolved to initiate infection by a unique mechanism.

## 1. Introduction

Human metapneumovirus (HMPV) was first isolated in 2001 in the Netherlands [1]. Dutch investigators discovered an unknown virus in respiratory secretions collected from young children with lower respiratory illness. Virus-infected cell supernatants were examined by electron microscopy and found to contain pleomorphic virus particles measuring 150 to 600 nm, with spike-like envelope projections of 13 to 17 nm [2]. PCR and sequence analysis revealed a single-stranded, negative-sense RNA genome with close resemblance to avian metapneumovirus (AMPV), an avian virus that causes serious respiratory disease in chickens and turkeys [3]. Based upon virion morphology and genome organization, HMPV was classified as the first human member of the *Metapneumovirus* genus, in the subfamily *Pneumovirinae* of the paramyxovirus family [1,4].

HMPV is a ubiquitous respiratory pathogen that has been circulating in human populations undetected for decades. The original report detected HMPV-specific antibodies in archived human sera from the 1950s [1] and HMPV has been detected by RT-PCR in specimens from 1976 [5]. Phylogenetic analysis of multiple HMPV gene sequences suggests that HMPV diverged from AMPV between 200–300 years ago [6,7]. HMPV is a leading cause of lower respiratory infection in infants and children worldwide [5,8,9,10,11,12,13,14,15,16,17,18]. HMPV is also associated with severe disease in immunocompromized hosts or persons with underlying conditions [19,20,21,22,23,24,25]. HMPV causes a clinical spectrum of illness from upper airway infection to severe lower respiratory tract infections (e.g., bronchiolitis and pneumonia) [5,26]. HMPV pathogenesis is similar to hRSV and causes inflammation, sloughing and necrosis of the bronchiolar epithelium [27]. Experimental studies in nonhuman primates and small animal models (hamsters, cotton rats, and mice) indicate that HMPV replicates in the upper and lower respiratory tract epithelium and demonstrate no evidence of viral dissemination, indicating a distinct tissue tropism for HMPV which is consistent with clinical illness observed during human infection [28,29,30].

## 2. HMPV F: A Dual Function Fusion Protein

**Figure 1:**
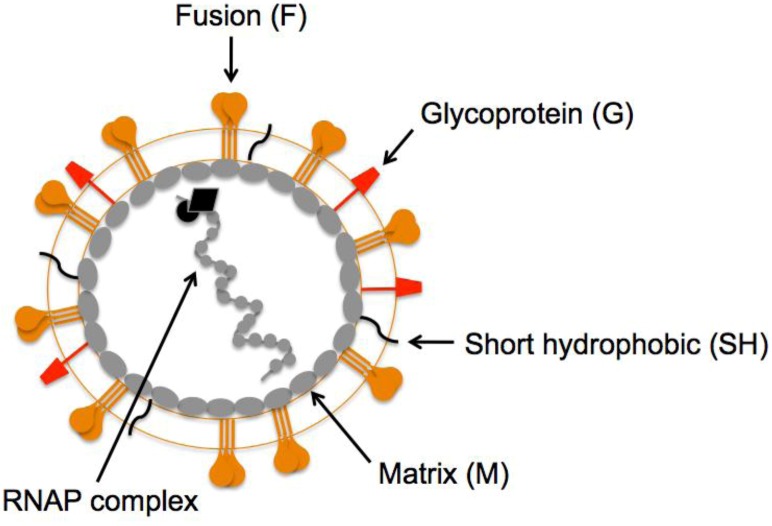
**Schematic representation of a human metapneumovirus (HMPV) virion. **The fusion (orange), attachment (red), and short hydrophobic (black) glycoproteins are depicted at the virion surface. The matrix protein (gray ovals) lines the inner leaflet of the virus membrane. Encapsidated within the viral envelope is the ribonucleoprotein (RNAP) complex consisting of the helical, genomic RNA wrapped by the nucleoprotein (N), the viral RNA-dependent, RNA polymerase (L), phosphoprotein (P), and matrix 2 protein (M2).

The HMPV virion is similar to other paramyxoviruses. The viral lipid bilayer is likely derived from the plasma membrane of infected cells during virus egress, as virions are thought to bud from the infected cell surface. The closely related pneumovirus, hRSV forms filaments at and buds from the apical surface of polarized epithelial cells [31,32]. Similar to hRSV [32], the HMPV matrix (M) protein has been shown to assemble into filaments on the surface of HMPV-infected cells [33]. The HMPV M protein lines the inner leaflet of the viral lipid bilayer, and virions contain three integral membrane surface glycoproteins, the fusion (F), glycoprotein (G) and short hydrophobic (SH) proteins (Figure 1). The envelope contains a helical ribonucleoprotein (RNAP) complex consisting of nucleoprotein (N), phosphoprotein (P), matrix 2 protein (M2), large polymerase protein (L), and the single-stranded, negative-sense RNA genome.

HMPV infection is initiated by viral surface glycoproteins that attach to cellular receptors and mediate virus membrane fusion with cellular membranes. Most paramyxoviruses use two viral glycoproteins to facilitate virus entry—an attachment protein, called HN, H or G (depending on the virus), and a fusion (F) protein. Indeed, elegant studies on paramyxoviruses in the subfamily *Paramyxovirinae* have demonstrated that viral attachment and fusion are mediated by two individual viral proteins that act in concert during virus entry (reviewed in [34]). For the *Paramyxovirinae*, F is capable of catalyzing membrane fusion only in the presence of the attachment protein. The proposed model for paramyxovirus entry is a stepwise process where: 1) the attachment protein binds to cellular receptors; 2) the bound attachment protein directly interacts with and transmits a signal to the F protein; 3) the F protein becomes activated to undergo structural changes; 4) F refolds from a prefusion to postfusion structural conformation, resulting in the merging of the viral membrane with the plasma membrane; and 5) the genome is delivered into the cytoplasm from a fusion pore created at the cell surface.

However, membrane fusion for paramyxoviruses in the *Pneumovirus* subfamily is unique in that F drives fusion in the absence of a separate viral attachment protein. The pneumoviruses incorporate three surface glycoproteins that could potentially facilitate entry: F, SH, and G (termed the attachment protein based upon homology to other paramyxoviruses). The first indication that pneumovirus entry may not absolutely depend upon all surface glycoproteins was the identification of a live, cold-passaged hRSV strain (cp-52) that had a large deletion spanning most of the SH and G coding sequences but replicated efficiently in Vero cells [35]. Thus, in the absence of SH and G, hRSV was infectious and capable of replicating to high titer *in vitro*, although cp-52 replication was severely attenuated *in vivo* in both animals and humans [35]. Subsequent studies with hRSV engineered without the SH and G genes have confirmed that hRSV F is sufficient for virus entry in cell culture, but replication is attenuated in animal models of infection [36,37,38,39,40,41,42]. The invention of reverse genetics for HMPV has enabled researchers to rescue viruses engineered to express the F protein in the absence of G and/or SH, and define the individual contributions of each protein for virus entry. Deletion of the SH gene from HMPV did not alter viral growth in cell culture or animal models of infection [43,44], confirming that the SH protein does not serve a role in virus entry. HMPV lacking both G and SH replicated efficiently in cell culture [44] and in both the upper and lower respiratory tracts of hamsters after intranasal inoculation [44]. HMPV lacking G (HMPVΔG) infected and was shed from both the upper and lower respiratory tracts of African green monkeys, a permissive nonhuman primate host. However, HMPVΔG replication was attenuated compared to wild type infection (viral titers were reduced six-fold in the upper respiratory tract and 3200-fold in the lower respiratory tract) [43].

The fact that both hRSV and HMPV viruses lacking the G and SH genes are infectious indicates that the F proteins from these pneumoviruses can perform the necessary functions for virus entry, *i.e.*, attachment and fusion. However, the G protein is clearly important for virus fitness *in vivo*. Despite the fact that hRSV and HMPV G proteins share no homology and are very different in size [4], the G proteins are thought to serve similar functions in the pneumovirus lifecycle. Current evidence suggests that pneumovirus G proteins help tether virus particles to the cell surface, and are likely important for strengthening particle adhesion and concentrating virions on the cell surface. Techaarpornkul *et al.* demonstrated that hRSV G is required for optimal virus attachment to the cell surface, but not for virus fusion and entry as the F protein alone is sufficient for efficient virus fusion with cultured cells [39]. HRSV and HMPV G have been shown to bind adhesion molecules on the cell surface such as heparan sulfate or glycosaminoglycans (GAGs) [45,46,47]. Pretreatment of HMPV with soluble heparin inhibits infection, and recombinant G protein binds to heparin-agarose columns and cells in a GAG-dependent manner [47]. Thus, HMPV G is capable of binding cell surface GAGs and may contribute to virus attachment; however, because G is dispensable for viral entry, it is not absolutely required for the membrane fusion activity of F during the virus entry process. 

Thus, HMPV F is necessary and sufficient for membrane fusion and capable of mediating entry without an additional attachment protein, in contrast to the firmly established model for most paramyxoviruses that absolutely require two viral proteins. To be sufficient for virus entry, HMPV F must be able to attach to cellular receptors and this attachment should activate F-mediated membrane fusion. How HMPV F mediates both attachment to cellular receptors and membrane fusion has been the focus of several recent studies and will be the topic of this review.

## 3. HMPV F: The Key to Intrusion

**Figure 2:**
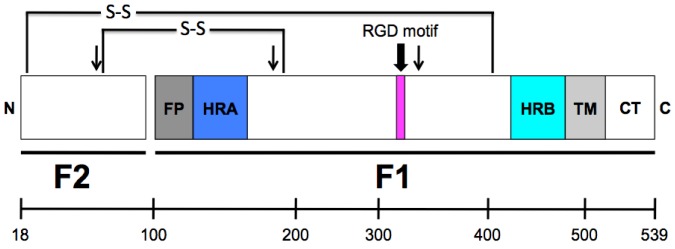
**Schematic representation of the cleaved HMPV fusion protein.** The mature, proteolytically cleaved HMPV F protein contains 522 amino acids (without signal sequence), with 82 residues in the F2 subunit and 440 residues in the F1 subunit, which includes a large extracellular domain, an ~23-amino acid transmembrane domain and a cytoplasmic tail of 25 residues. FP = fusion peptide; HRA = heptad repeat A; HRB = heptad repeat B; TM = transmembrane domain; CT = cytoplasmic tail. The approximate location of a conserved RGD motif (residues 329–331) is indicated as a magenta box. Arrows indicate the three N-linked glycosylation sites. The location of two disulfide bonds that connect the F1 and F2 protein subunits are shown. (Scale bar, amino acids.)

All paramyxovirus F proteins are class I fusion proteins that due to the conservation of structural domains are thought to mediate fusion via the same global mechanism (reviewed in [48]). HMPV F is a trimeric, type I membrane glycoprotein. A schematic of the HMPV F protein structure is shown in Figure 2. Each F monomer is translated as an inactive precursor, F0, which is proteolytically cleaved at a monobasic consensus site by host cell proteases into two disulfide-linked subunits, F1 and F2. This cleavage reveals a hydrophobic fusion peptide (FP) at the N-terminus of the larger F1 subunit, which inserts into target cell membranes to initiate membrane fusion. Two heptad repeat (HR) regions are located in the F1 subunit; HRA is adjacent to the FP and HRB is adjacent to the transmembrane (TM) domain. During fusion a metastable, prefusion conformation of F refolds into a highly stable postfusion conformation. During the prefusion-to-postfusion transition, the HRA and HRB form trimeric coiled-coils that rearrange to fold into a highly stable six-helix bundle that drives the formation of a fusion pore between the virus and cell membranes. Formation of the six-helix bundle is essentially irreversible and is directly linked to membrane merging, as peptides mimicking the heptad repeats are capable of blocking membrane fusion [49,50,51].

**Figure 3:**
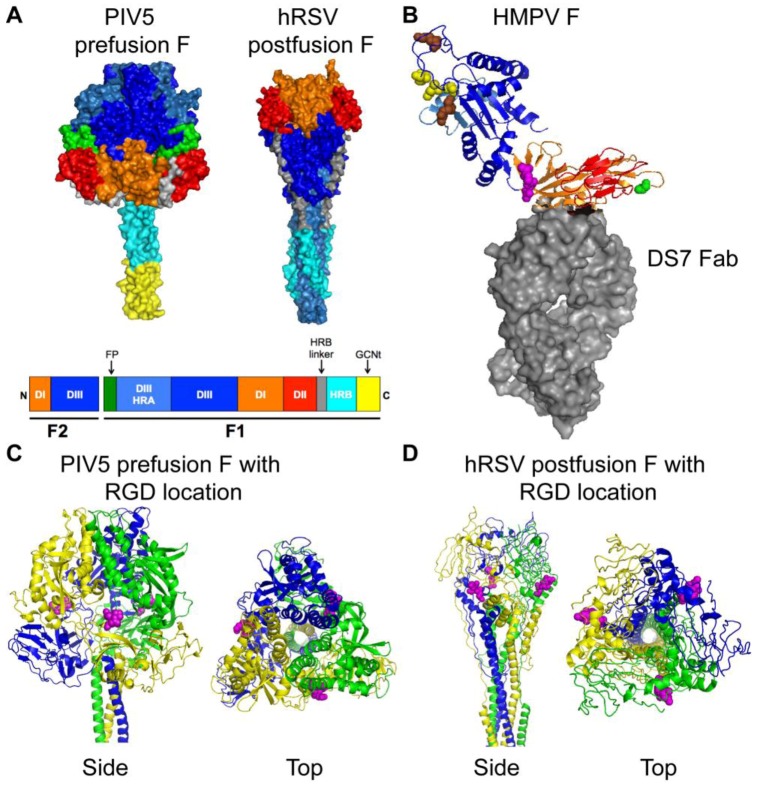
**Structures of paramyxovirus F proteins.** (**A**) The prefusion conformation of the PIV5 F trimer and the postfusion conformation of the hRSV F trimer. A schematic of the F protein color-coded to identify structural domains is shown below the structures. The DI domain is shown in orange, the DII domain is shown in red, and the DIII domain is shown in blue. HRA is shown in light blue, HRB is shown in cyan and the HRB linker domain is shown in gray. The fusion peptide is shown in green for prefusion PIV5 F, but was not solved in the hRSV F structure. The GCNt domain (shown in yellow) was added to the PIV5 F trimer for crystallization. (**B**) HMPV F in complex with a neutralizing antibody (DS7 Fab, shown as gray surface). DI, DII, and DIII coloring is as for the F trimers shown in (**A**). The conserved RGD motif is shown as magenta spheres. Residue 294 (Glu) is shown as green spheres. Conserved charged residues in DIII that impact fusion activity are indicated as spheres (yellow, acidic residues; brown, basic residues). (**C**) Side and top views of the prefusion PIV5 F trimer are shown with the homologous residues to HMPV F-RGD shown as magenta spheres. Each monomer is shown in blue, yellow, or green. (**D**) Side and top views of the postfusion hRSV F trimer are shown with the homologous residues to HMPV F-RGD shown as magenta spheres. Coloring is as for PIV5 F in (**C**). Structural coordinates were obtained from the Protein Data Bank [52] and figure constructed with PyMOL. PIV5 F, PDB ID: 2B9B [53]; hRSV F, PDB ID: 3RKI [54]; HMPV F complex, PDB ID: 4DAG [55].

Crystal structures for the PIV5 prefusion F [53] and the hRSV postfusion F [54] have been determined and are shown in Figure 3A. Three discrete domains, DI (orange), DII (red) and DIII (blue), have been colored to indicate how the linear sequence of amino acids is arranged in the tertiary structure of the prefusion and postfusion F proteins. The HRA domain (light blue) changes significantly in both secondary structure and location, and the F head domain becomes more compact, during the prefusion-to-postfusion F transition (reviewed in [48]). A crystal structure of HMPV F in complex with a neutralizing antibody (DS7 Fab) has recently been described [55]. The HMPV F structure was solved as a monomer, and is depicted in Figure 3B. The HMPV F DIII domain is similar to the prefusion PIV5 F protein but overall the HMPV F structure resembled the hRSV postfusion F [55].

HMPV F amino acid sequences are highly conserved, falling into two major lineages (A and B), each with two subgroups (1 and 2), which exhibit a mean of ~96% identity [7,56]. A study in 2004 comparing 64 partial F gene sequences from the United States, Canada, Peru, France, Israel, Republic of South Africa, Australia and the Netherlands confirmed that HMPV isolates circulating around the world encode F proteins from the A1, A2, B1 and B2 genetic lineages [57]. Distinct canonical amino acid differences are present between major subgroups, and polymorphic variations tend to cluster in discrete regions [7,57]. Amino acid identity within and between subgroups is higher than nucleotide identity, suggesting structural or functional constraints on F protein diversity. A study comparing 85 full-length F gene sequences collected over a 20-year period from the United States (all isolated in Tennessee), Canada, Japan and the Netherlands suggested that there was no progressive genetic drift over time, and the genetic lineages were stable over time in circulating viruses in a population of children with respiratory illnesses [7].

The HMPV F protein is glycosylated at three Asn residues (residues 57, 172 and 353) [58,59]. Mutating the F2 subunit glycosylation site at N_57_ results in an F protein that is expressed and processed at near wild type levels, and efficiently mediates both cell-cell fusion [58] and virus growth in a mouse model of infection [59]. Glycosylation of N_172_ appears to be critical for proper F protein folding, fusion activity, and virus growth in mice [58,59]. Glycosylation of N_353_ appears to be critical for proper F folding and cleavage [58], which results in the inability to recover viruses with an N353Q mutation [59].

An arginine-glycine-aspartate (RGD) motif at residues 329–331 in the HMPV F1 subunit, along with 5 N- and 16 C-terminal flanking residues, is strictly conserved in all gene sequences that have been studied to date, regardless of genetic lineage and despite diversity in other regions of the F gene [60]. This motif is unique to HMPV F among human paramyxovirus fusion proteins and absent in all published HRSV F sequences. The RGD motif is located in DI of the HMPV F protein and is shown in Figure 3B as magenta spheres. Based upon homology, the predicted location of the RGD motif is also shown on the PIV5 prefusion (Figure 3C, magenta spheres) and the hRSV postfusion (Figure 3D, magenta spheres) F structures. The presence of the invariant RGD motif led to the speculation that integrins function as receptors for HMPV, discussed in more detail in the next section.

## 4. HMPV Binding: Identifying the Door

As noted, HMPV diverged from an avian pneumovirus (AMPV-C) between 200–300 years ago [6,7]. HMPV does not replicate in birds and evidence of AMPV infection has not been detected in humans [1]. The switch in virus tropism appears to be due to the attachment function of the F protein. In cell culture, HMPV expressing only F (HMPVΔGΔSH) binds to the cell surface with the same efficiency as wild type virus [61]. Moreover, HMPV F confers species specificity of infection, as F was found to be primarily responsible for the difference in the ability of AMPV-C and HMPV to infect quail fibroblast (QT6) cells [62]. Further, the F2 subunit (and not the G protein) of both hRSV [38] and HMPV [62] has been shown to confer species-specific infection of cells. This evidence strongly indicated that HMPV F interacts with cell surface receptors, and both protein and carbohydrate receptors for HMPV F have been discovered. 

We identified a conserved RGD motif in the HMPV F1 subunit, and speculated that integrins might serve as receptors for HMPV. Integrins are heterodimeric integral membrane proteins composed of one α and one β subunit. A subset of integrins (αVβ1, αVβ3, αVβ5, αVβ6, αVβ8, α5β1, α8β1 and αIIbβ3) bind proteins with RGD motifs, such as fibronectin and vitronectin. Integrins are adhesion receptors that bind extracellular proteins to modulate cell behavior and survival. Integrins associate with cytoskeletal proteins, adaptors, and kinases via the cytoplasmic tails of the α and β subunits, allowing them to transduce bidirectional signals between the intra- and extra-cellular environments [63]. The intimate link between integrins and cell signaling cascades, as well as endosomal sorting pathways, makes them a desirable receptor for mammalian viruses. Several viruses, including adenovirus, hantavirus, herpesvirus, picornavirus, and reovirus, utilize integrins during entry, either as attachment or internalization receptors (reviewed in [64]).

Initial experiments in our laboratory found that the RGD-binding integrin αVβ1 promoted HMPV infection [60,65]. EDTA, which chelates divalent cations required for integrin function, exhibited a dose-dependent inhibition of HMPV infection. Synthetic RGD peptides blocked HMPV infection, while RGE peptides did not. Function-blocking monoclonal antibodies (mAbs) directed against RGD-binding integrins inhibited HMPV infection, with mAbs against αV and β1 exhibiting the most potent effect. Similarly, reduction of αV and β1 expression by siRNA reduced HMPV infection. Importantly, none of these loss-of-function experiments inhibited hRSV, which has a very similar F protein but lacks an RGD motif. Transfection of poorly permissive cells with αV or β1 cDNAs conferred HMPV infectivity. These data suggested that αVβ1 integrin is a receptor for HMPV.

Subsequent studies using both virus and virus-like particles showed that HMPV F is capable of binding multiple RGD-binding integrins in addition to αVβ1, specifically α5β1 and other αV-integrin heterodimers that may differ depending upon target cells [65]. Furthermore, recent data suggest that HMPV F utilizes RGD-binding integrins as attachment and entry receptors. HMPV F binds RGD-binding integrins during attachment, and this interaction is necessary for virus attachment and subsequent productive infection [65]. Blockade of integrin function with mAbs inhibits approximately half of virus binding, but >90% of virus infectivity. Thus, while HMPV can bind to other cell surface molecules, integrin binding is required for efficient entry. Furthermore, mutating the conserved RGD motif significantly attenuated HMPV growth *in vitro* and altered the extent of virus-infected cell-cell fusion, suggesting a key role for the integrin-binding motif during F-mediated entry and fusion [65]. In addition to being necessary for efficient HMPV F-mediated attachment, our experiments suggest that integrin engagement is also required for postbinding events during HMPV entry, because blocking RGD-binding integrin attachment results in significantly lower levels of viral transcripts at eight hours post infection [65]. Thus, in the absence of integrin-mediated entry, virus transcription is impaired. These data suggest that RGD-binding integrin engagement is necessary but not sufficient for HMPV attachment (40–50% of HMPV F-specific binding is mediated by integrins), and that integrin engagement is important for post-binding events that occur during HMPV entry [65].

Chang *et al.* recently identified heparan sulfate as a receptor for HMPV F, and suggested that the carbohydrate moiety was the first binding partner for HMPV F during virus attachment [61]. Experiments with Chinese hamster ovary (CHO-K1) cells and mutant CHO-K1-derived cell lines unable to synthesize GAGs demonstrated that heparan sulfate proteoglycans were required for HMPV binding and infection [61]. These authors concluded that β1 integrin was not a direct cellular receptor for HMPV because virus still bound β1 integrin-deficient murine embryonic fibroblasts [61]. However, when β1-null fibroblasts were complemented with human β1 integrin, HMPV infection was significantly enhanced, supporting a role for β1 integrin in promoting HMPV infection [61]. In light of our recent findings that HMPV F binds multiple integrins during attachment, HMPV F would be expected to bind to other RGD-binding integrins such as αVβ5, αVβ6 and/or αVβ8 on the β1-null cell surface. The β1-null fibroblasts express several αV heterodimers, including αVβ3 and αVβ5 [66,67]*.* These data show that an HMPV F interaction with heparan sulfate moieties on the cell surface contributes to HMPV attachment and infection, and heparan sulfate may be the first cell surface receptor that HMPV engages during virus attachment. 

Interestingly, recent findings by Chang *et al. *suggest that efficient HMPV infection depends upon the expression of a proteinaceous receptor that is trypsin- and proteinase K-sensitive (integrins are reported to be resistant to both types of protease treatment) [61]. Thus, HMPV entry appears to involve more than one cell surface receptor: heparan sulfate, RGD-binding integrins, and other protease-sensitive surface proteins. However, further studies are needed to identify other putative proteinaceous receptors for HMPV.

Together, recent studies on the receptors involved in HMPV entry suggest that HMPV F interacts with multiple binding partners during attachment. We propose a multi-step model of HMPV attachment where F first binds heparan sulfate before engaging specific proteinaceous receptors including RGD-binding integrins, which mediate post-binding events that lead to productive HMPV infection.

## 5. HMPV Fusion: Entering the Premises

The mature HMPV F protein must be proteolytically cleaved to convert the F0 precursor into the fusion-competent, disulfide-linked F1/F2 heterodimer. The monobasic, consensus cleavage site (PRQSR) is a trypsin-like cleavage motif, which is cleaved after the final Arg residue. For most cell types, cell surface HMPV F is expressed in the F0 precursor form. In cell culture, F protein cleavage occurs when trypsin is added to the cell medium. Indeed, HMPV grows poorly (or not at all) unless trypsin is added to virus growth medium during virus propagation in cell culture [1,44,68]. Trypsin treatment of HMPV F-expressing cells is also required for cell-cell fusion activity [58]. *In vivo*, cleavage is likely mediated by extracellular proteases such as TMPRSS2 [69] or mini-plasmin [70] expressed in the respiratory tract of infected humans. HMPV F cleavage activates the protein by creating a new F1 N-terminus, the fusion peptide, which is inserted into target cell membranes during the fusion process.

Most paramyxovirus F proteins require an attachment protein to drive membrane fusion, both during entry (virus-cell fusion) and to induce syncytia (cell-cell fusion). Transient transfection of HMPV F is sufficient to induce cell-cell fusion, and co-expression of the G protein does not significantly enhance fusion [58]. Moreover, using virus-like particles containing only F or F+G, we have demonstrated that the G protein does not alter particle-cell hemifusion kinetics or the extent of fusion [65]. This evidence coupled with the observation that HMPV lacking the G protein is infectious suggests that the G protein is not a critical component of the HMPV fusion machinery. 

To better understand how the HMPV F protein functions alone to mediate fusion, recent studies have explored the requirements for fusion and investigated the importance of specific residues within the extracellular domain of the protein for fusion activity. Paramyxovirus fusion proteins mediate fusion in a pH-independent manner [71], and commonly induce fusion of cultured cells at neutral pH resulting in syncytium formation during virus infection. Surprisingly, the first report of HMPV F-mediated cell-cell fusion activity suggested that HMPV F required low pH for activation [58]. Schowalter *et al.* determined that HMPV F required both trypsin treatment, to mediate proteolytic processing, and low pH pulses for fusion activity of the A2 genotype strain CAN97-83 [58]. A follow-up study suggested that the low-pH-induced fusion phenotype of HMPV CAN97-83 may be strain-specific [72]. Herfst *et al.* used prototype F proteins from the four HMPV genetic lineages to demonstrate that fusion for lineage B strains was pH-independent, but fusion for some lineage A strains (one A1 and one of two A2 strains tested) was enhanced by pH 5 pulses [72]. Mutagenesis studies determined that a Gly residue at position 294 was a key determinant of the low-pH-induced fusion phenotype for HMPV A strains, but inserting the Gly_294_ residue into HMPV B F proteins did not confer pH sensitivity [72]. We compared the sequences of more than 1000 published full-length and partial HMPV F sequences from around the world to determine the frequency of the residue present at position 294 (see Figure 3B, green spheres). Most HMPV strains encode a Glu residue at position 294, 6% encode a Gly (59 of 1005), and rarely a Lys residue is found at this position in the sequence (15 of 1005). The lack of HMPV F proteins with the low-pH sensitive Gly_294_ suggests that exposure to low pH is not a general requirement for fusion activity. Thus, the general trigger that activates HMPV F to drive the membrane fusion process is still not known.

The finding that some HMPV F strains may require low pH for fusogenicity has led to follow-up studies that have elucidated the importance of other residues in the HMPV F protein that serve a role in protein stability and fusion triggering. Mutagenesis studies of CAN97-83 suggest that protonation of a conserved His residue at position 435 in the HRB linker domain serves a critical role in the low pH activation of the F protein [73,74]. A predicted cluster of basic residues Lys_295_, Arg_396_, and Arg_438_ are also important for the low pH fusion phenotype [73]. Interestingly, a predicted cluster of positively charged residues in this same region of the AMPV-A F protein (a distantly related pneumovirus) are important for fusion activity at neutral pH [75]. These studies suggest that the His residue at position 435 may serve as a low-pH sensor, and one model proposed that protonation of the His residue at low pH induces localized electrostatic repulsion that leads to the destabilization of F and fusion triggering [73,76]. Alternatively, electrostatic repulsion among the basic residues induced by receptor binding could destabilize the HRB linker domain and trigger fusion [75]. In any case, mutational analyses suggest that specific residues in domain II of HMPV F (Figure 3B, red residues) impact the initiation of membrane fusion. It is interesting that the cluster of basic residues is located in close proximity to a recently identified epitope that is recognized by a potently neutralizing mAb [55,77]. Thus, structural and functional studies support a significant role for domain II in the fusion activity of HMPV F.

Another charged region in the HMPV F extracellular head domain has recently been implicated in the low-pH sensitive fusion phenotype. Acidic residues Glu_51_, Asp_54_, and Glu_56_ (Figure 3B, yellow spheres) are important for low-pH triggered F-mediated cell-cell fusion, but also appear to be critical for protein stability [76]. These residues are clustered in a charged region present in the F2 subunit of F in close proximity to two basic residues (Figure 3B, brown spheres) [76]. The specific acidic residues in the F2 subunit and basic residues in the HRA domain are highly conserved in all HMPV strains; therefore, others have predicted that salt bridges and/or electrostatic interactions in this region may contribute to F triggering during HMPV fusion [76]. This hypothesis is supported by another study that indicated that the F2 subunit from AMPV-C could confer a hyperfusogenic phenotype to HMPV F1 [62].

Taking these studies together, it is clear that multiple regions in the HMPV F head domain are critical for fusion activity. Interestingly, two distal regions with predicted charged residue clusters have been implicated in fusion triggering (located in domains II and III). The idea that electrostatic repulsions can trigger the HMPV F prefusion protein to spring into the postfusion conformation during the fusion process is logical. The HRA region of F must undergo a significant structural rearrangement during fusion, extending to form a long coiled-coil and moving a long distance to refold into a six-helix bundle with HRB coils near the TM domain [48]. This structural change must also occur at the right time and place, as refolding of class I fusion proteins is irreversible under physiological conditions. Thus, sensor residues could act as molecular switches that modulate electrostatic interactions, leading to repulsive forces that drive movement of structural elements within the F protein. While histidine protonation at residue 435 appears to serve as one sensor in HMPV strains that exhibit enhanced fusogenicity at low pH, it remains unclear how pH-independent HMPV F fusion is triggered. Perhaps receptor interactions serve to augment local protein structure, driving the same electrostatic repulsion that can be triggered by exposure to low pH in some HMPV strains.

It is tempting to speculate that a direct interaction between HMPV F and RGD-binding integrins serves to initiate F-mediated fusion. However, current evidence does not support this hypothesis. We recently found that blocking HMPV F interaction with RGD-binding integrins did not alter virus-cell hemifusion kinetics, and virus-like particles bearing an F-RAE mutation fused with the same hemifusion kinetics as wild type F particles [65]. Moreover, Chang *et al.* showed that an HMPV F-RGA mutant promoted cell-cell fusion at levels approximately 80% of the wild type F protein [61], indicating that the RGD motif was not absolutely required for fusion activity. However, our results support a post-binding role for RGD-binding integrins during HMPV entry, as integrin blockade reduced HMPV transcription at 8 hours by 50%, in addition to a 40–50% block during virus attachment [65]. Further, when we introduced the F-RAE mutation into a virus, the F-RAE virus was severely attenuated and exhibited a small plaque phenotype that lacked the characteristic syncytia (indicative of F-mediated cell-cell fusion) of wild type virus [65]. Thus, while it appears that an F-integrin interaction is not required for efficient HMPV hemifusion, it remains possible that this interaction promotes fusion pore opening during virus-cell fusion.

## 6. HMPV Entry: Unlocking the Mystery

The solved crystal structures of postfusion PIV3, NDV, and hRSV F proteins are similar [54,78,79], supporting the model that all paramyxovirus F proteins mediate fusion via a highly conserved mechanism. Only one paramyxovirus F protein, PIV5, has been crystallized in the prefusion structural conformation (shown in Figure 3A, [53]). Comparison of this structure to the postfusion conformation has facilitated our understanding of the paramyxovirus fusion process (reviewed in [48]). The unique activity of pneumovirus fusion proteins to mediate fusion in the absence of an attachment protein suggests that hRSV and HMPV F may have unique prefusion structures compared to the *Paramyxovirinae* subfamily F proteins. Unfortunately, crystal structures of hRSV and HMPV prefusion trimers have not been published. The recently described structure of HMPV F in complex with a neutralizing antibody [55] provides insight into the structure of the protein, but HMPV F may trimerize slightly differently than PIV5 F [53]. Furthermore, while the HMPV F DIII domain had a similar fold to the PIV5 prefusion protein, the overall structural fold more closely resembled the postfusion hRSV F protein [55]. Thus, a crystal structure of a prefusion pneumovirus F trimer will be required to understand how these proteins have evolved to mediate fusion in the absence of an attachment protein. Further, because hRSV and HMPV F proteins utilize distinct proteinaceous cellular receptors [65,80], these proteins may also have unique prefusion structures. Such structures could provide insight into how pneumovirus F proteins couple receptor-binding and fusion activities.

The proverbial “black box” with respect to paramyxovirus entry is a lack of understanding of the fusion protein triggering process. For all paramyxoviruses, the process by which receptor binding triggers conformational changes in F remains unclear. HMPV F interacts with at least two different cell surface receptors during entry, heparan sulfate and RGD-binding integrins. How binding to receptor(s) results in structural changes in F that drive fusion is not clear. Furthermore, whether HMPV binds receptors simultaneously or sequentially is not known. Simultaneously binding to multiple receptors may induce conformational changes in multiple sites of the HMPV F extracellular domain, thereby promoting destabilization, protein refolding, and fusion. Alternatively, HMPV F binding to one receptor may expose the receptor binding site for another co-receptor, similar to the strategy utilized by HIV gp120/gp41 during fusion [81]. Our understanding of HMPV F triggering is further complicated by observations that some F proteins are more fusogenic at low pH. Typically, enveloped viruses either fuse at neutral pH or require exposure to low pH for fusion activity. We speculate that low pH treatment could result in histidine protonation events that serve to destabilize HMPV F in a similar manner to receptor engagement, although this hypothesis requires further investigation. This could explain why not all strains require low-pH exposure, but highly conserved charged regions of F are critical for function of all HMPV strains. 

The role of low pH in HMPV entry is still unclear. Inhibitors of endosomal acidification such as bafilomycin A1 and concanamycin A have been shown to partially reduce HMPV infection of the low-pH sensitive CAN97-83 virus strain [73]. Intriguingly, bafilomycin A1 and concanamycin A also partially inhibit infection by the pH-independent NL199 (B1) strain of HMPV, but not the NL1-00 (A1) strain which has been shown to be triggered by low pH exposure in syncytium assays [74]. Because the low-pH-sensitive phenotype observed in cell-cell fusion assays does not necessarily correlate with inhibition of HMPV infection by endosomal acidification inhibitors, it is not clear that the ability to enhance fusogenicity with low pH pulses is correlated with a requirement for endosomal acidification during entry. Furthermore, whether particle-cell fusion requires exposure to low pH requires further investigation to determine whether low pH is an absolute requirement for triggering of F proteins from some HMPV strains. The site of HMPV fusion also requires further investigation. Schowalter *et al.* reported that infection by the low-pH sensitive HMPV strain CAN97-83 was significantly impaired by chemical inhibitors of endocytosis pathways, e.g., chlorpromazine and dynasore [73]. Thus, one study suggests that HMPV entry may involve virus internalization, although this speculation requires further studies with other HMPV lineage strains and more specific inhibitors of various endocytosis pathways.

## 7. Conclusions

HMPV is a leading cause of lower respiratory illness in adults and children. Although the virus was only discovered in 2001, remarkable progress has been made in elucidating the biology of HMPV. The F protein serves both to bind cellular receptors and to mediate fusion, although G protein is required for full virulence. HMPV F binds to heparan sulfate, and uniquely among human paramyxoviruses uses multiple RGD-binding integrins as attachment and entry receptors. Some HMPV F molecules exhibit sensitivity to low pH, though the function of this low pH triggering during the context of infection is not clear. While HMPV F shares many features of other paramyxovirus fusion proteins, there are distinct aspects of the attachment, entry, and fusion mechanisms of this recently discovered virus.
